# Crystallization Behavior and Crystallographic Properties of dl-Arabinose and dl-Xylose Diastereomer Sugars

**DOI:** 10.1021/acs.cgd.1c01329

**Published:** 2022-01-12

**Authors:** Bradley Tyson, Christopher M. Pask, Neil George, Elena Simone

**Affiliations:** † School of Chemical and Process Engineering, University of Leeds, Leeds LS2 9JT, United Kingdom; ‡ School of Chemistry, University of Leeds, Leeds LS2 9JT, United Kingdom; § Syngenta Jealotts Hill Int. Research Centre, Bracknell, Berkshire RG42 6EY, United Kingdom; ∥ School of Food Science and Nutrition, Food Colloids and Bioprocessing Group, University of Leeds, Leeds LS2 9JT, United Kingdom; ⊥ Department of Applied Science and Technology (DISAT), Politecnico di Torino, 10129 Torino, Italy

## Abstract

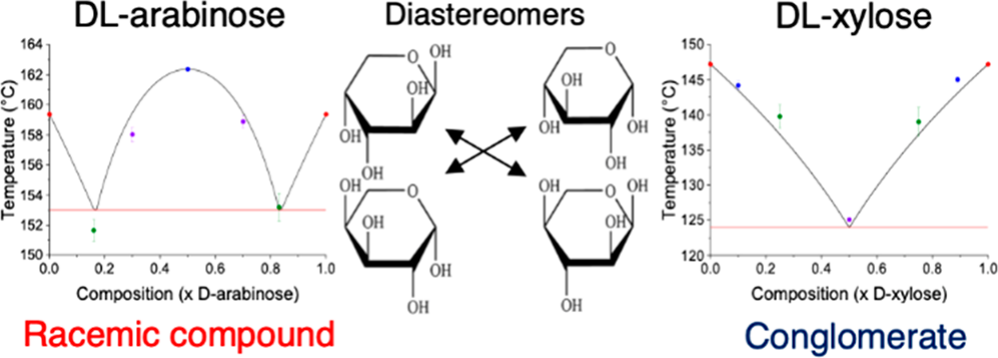

Natural sugar molecules such as xylose and arabinose exhibit sweetness profiles similar to sucrose, which makes them a valuable alternative in low-calorie foods as well as excipients or cocrystallization agents in pharmaceutical formulations. Xylose and arabinose are also chiral diastereomers that can exhibit specific crystallization behavior. In this work, the solid-state landscapes of the chiral pairs of both xylose and arabinose have been investigated to determine whether racemic compounds or conglomerates are formed. Furthermore, single crystals of xylose and arabinose have been grown and characterized by X-ray diffraction and optical microscopy to study their crystallographic properties and relate them to the crystallization behavior. Differential scanning calorimetry (DSC) measurements were used to determine the phase diagrams of the two analyzed chiral systems. The solubilities of the different solid forms of xylose and arabinose were measured in different solvent mixtures by a thermogravimetric method. An analysis was conducted to assess the main thermodynamic parameters and the activity coefficients of the compounds in solution. Finally, slurry experiments in a 50:50 w/w ethanol/water solvent have also been performed to determine the relative stability of each solid form and the kinetics of transformation in this solvent mixture. It was found that dl-arabinose crystallizes as a stable racemic compound, which transforms quickly from its constituent enantiomers when in solution; whereas d- and l-xylose molecules crystallize separately as a conglomerate.

## Abstract 2

The crystallization behavior of two chiral sugars, xylose and arabinose, is shown. Xylose forms a conglomerate and arabinose a racemate. The crystal structures of the different solid forms are reported here, together with their solubility in mixtures of water and ethanol and the binary phase diagrams of each enantiomeric pair, estimated from differential scanning calorimetry.

## Introduction

Sugar molecules are used extensively in the pharmaceutical and food industry mainly due to their properties as sweeteners or taste maskers.^1,2^ The most commonly used sugar for the purposes of sweetening is the disaccharide sucrose.^3,4^ Though it is used ubiquitously, concerns for diets containing high levels of sucrose have long been present.^5−9^ A current trend is the use of natural sugars or artificial sweeteners due to their high sweetness but lower caloric content and glycemic response.^1,10,11^ Natural sugars are preferable to artificial sweeteners as they can be extracted from plants, seeds, or milk without the use of organic solvents and, in some cases, using green technologies. Furthermore, natural sugars are nontoxic and fully biocompatible, and they are classified as a food ingredient rather than food additives. Two monosaccharides of recent interest are arabinose and xylose, which find many uses as alternatives to artificial sweeteners.^1,4,10,12^l-Arabinose is also an intermediate for drug synthesis, but it has been primarily studied for its use as a food ingredient to control calorie intake.^1,10,13^ The naturally occurring d-xylose, also known as wood sugar, is used as a sweetener and in the production of xylitol, which is commonly found in sugar-free chewing gum.^14−16^

Aldopentose sugars such as xylose and arabinose exhibit chiral behavior since these molecules have four asymmetric centers. Xylose and arabinose each exist as a d- and l-enantiomer, mirror-like molecules that have the same chemistry and physical properties but might present different biological activities.^17,18^ Arabinose is isolated as the natural form l-arabinose, but the d enantiomer can be synthesized through enzymatic pathways from other sugars such as d-xylose.^19^l-Xylose is also commonly synthesized through enzymatic pathways from other sugars since it does not exist in nature.^20^ Both enantiomers of xylose and arabinose find multiple uses in the food and pharmaceutical industries, as well as for the synthesis of other chemicals.^21−23^

Crystallization is the most common unit operation used to recover both d- and l-enantiomers of xylose and arabinose after enzymatic reactions, though additional purification steps are usually required prior to crystallization to ensure high product purity.^24^ Nevertheless, these diastereomers are often crystallized together or in the presence of multiple impurities that can affect the rates of nucleation and growth; for example, mannose can decrease the growth rate of d-xylose crystals of up to 30%, at a relatively low concentration.^25^ Seeding can help with the isolation of a specific diastereomer from a mixed solution,^26−28^ but a good knowledge of the solubilities of the different diastereomers and how they are affected by solvent composition is essential to improve the yield of recovery and the purity of the final product. Furthermore, both xylose and arabinose are known to form cyclic structures in solution, with multiple conformations known as anomers.^3,40,42−44^ These anomers and cyclic structures are formed in solution via dynamic ring opening and closing reactions, through their straight chain sugar intermediates. Such solution equilibria will have an effect on the crystallization behavior of xylose and arabinose, as only few of these existing anomers and cyclic structures might be present in the existing solid forms of the two sugars.^45^ In fact, regardless of the solvent from which they were crystallized, the previously reported d-xylose crystal structures present only molecules of the α-anomer of the six-membered ring structure, while l- and dl-arabinose crystals consist only of the β-anomer of the same type of ring conformation.^40−42,46−48^ In this work, the link between solution equilibria and crystallization behavior of xylose and arabinose will be analyzed; this knowledge can ensure efficient recovery and purification of these materials.

Furthermore, understanding crystallographic properties such as crystal habit and solid form landscape for these diastereomer systems is required for the formulation of products containing these sugars. Chiral molecules can have interesting crystallization behavior from solutions where both enantiomers are present. When both enantiomers crystallize together within the same unit cell, the crystal structure formed is known as a racemic compound; whereas when the two enantiomers crystallize separately they form a conglomerate.^17,29,30^ In other cases, a solid solution may be formed where one enantiomer is distributed within the lattice of the other.^29^ In order to design effective separation processes and ensure enantiomeric purity of the final product, it is essential to understand how enantiomers crystallize. In fact, while the two enantiomers of conglomerate systems might be separated by preferential crystallization from the same solution, additional unit operations might be required for efficient separation of enantiomeric pairs that form racemates or solid solutions.^31−37^

Structural differences between racemic compounds and conglomerates are identifiable by X-ray diffraction.^38,39^ Experimental studies have previously shown evidence that that dl-arabinose system crystallizes as a racemic compound, while it is unclear how mixtures of d- and l-xylose behave.^40,41^ Racemic compounds usually have different crystal structures, and they present different solubility and thermal properties compared to their enantiomers or to conglomerate pairs.^29,49^ A racemic compound could show the same, higher, or lower melting point than its pure enantiomers, whereas a conglomerate would always show a lower melting temperature.^17,29,30,50,51^ In any case, the use of X-ray diffraction is an essential technique for analyzing chiral systems.^38,39^

As arabinose and xylose are diastereomers, there will be differences in the solid-state physical properties between the two species,^17,52^ including solubility. Reliable solubility measurements are important for the design of unit operations for the efficient separation of arabinose and xylose from mixtures of these two diastereomers, which are a common result of some of their extraction processes.^25,53−55^, Sugar molecules usually have high water solubility and poor alcohol solubility, as such the use of mixed solvent systems is often employed to reduce the liquid viscosity and the solid loading in sugar crystallization in order to facilitate this unit operation.^56−59^ It has been reported that d-xylose has a higher solubility than l-arabinose in mixed solvent systems of methanol/water and ethanol/water, but as of yet reliable solubility data, including those of the possible racemic compounds, are still missing.^57,58^ Solubility data in different solvents are also important to determine the relative stability of the different solid forms of xylose and arabinose diastereomers and to estimate the kinetics of transformation toward the most stable form.^60^

In summary, here the solid-state landscapes of dl-arabinose and dl-xylose and the relative solubilities and thermodynamic properties of the different solid forms have been investigated. The results presented in this work can help in the design of efficient crystallization processes for the separation of xylose and arabinose diastereomers from mixed solutions, as well as in the identification of the correct sequence of processes for the isolation of either xylose or arabinose enantiomers from their respective racemic solutions.

## Materials and Methods

**Figure 1:**
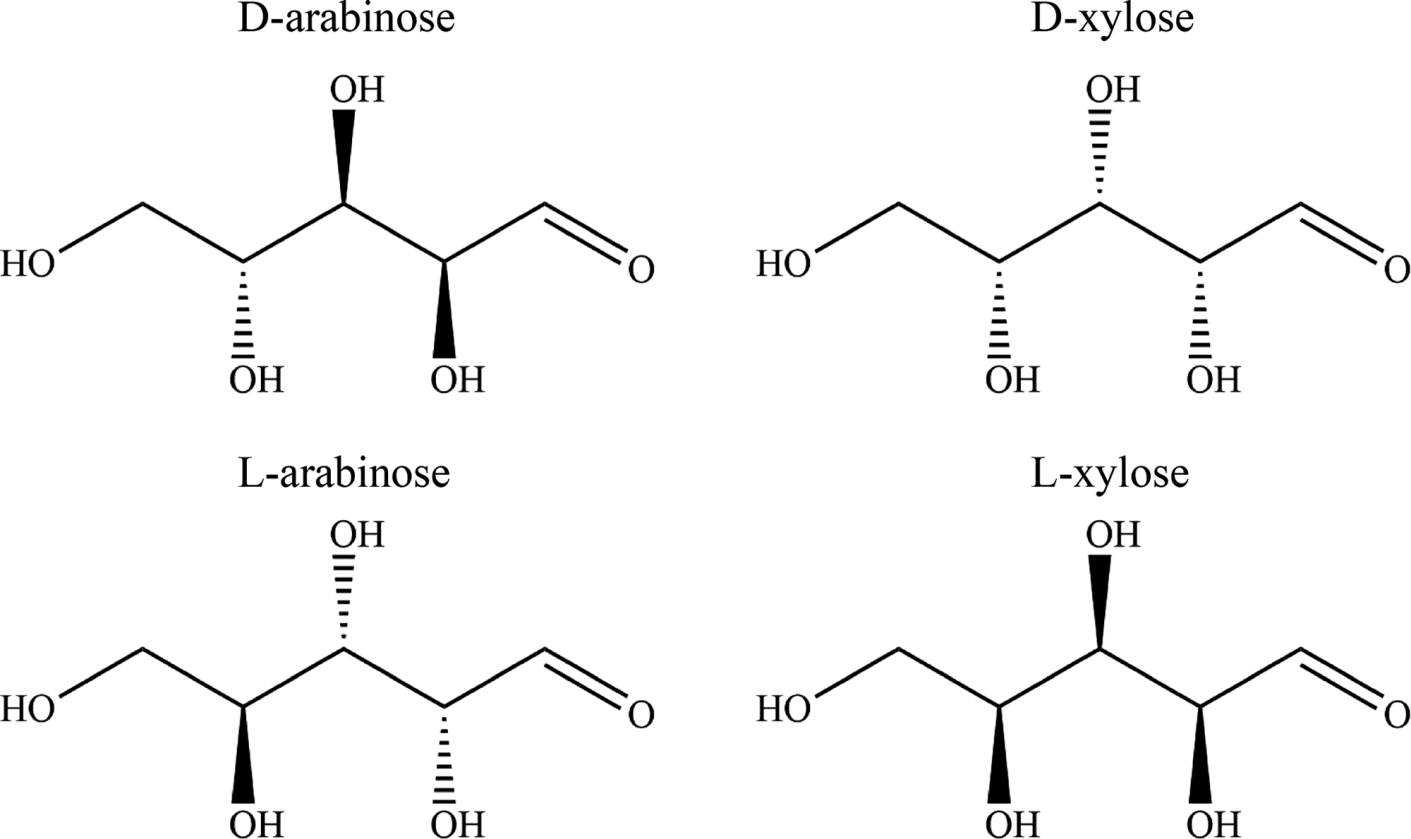
Structural representations of the two enantiomer pairs of dl-arabinose and dl-xylose used in this study.

d-Xylose (95% purity) was purchased from Fluorochem (Glossop, United Kingdom). d-Arabinose (99% purity) and l-arabinose (99% purity) were both purchased from Apollo Scientific (Manchester, United Kingdom). l-Xylose (99%) was purchased from Fisher Scientific (Loughborough, United Kingdom). Ethanol (absolute) purchased from VWR (Lutterworth, United Kingdom) and distilled water were used as solvents for the experiments. A representations of the solid materials used in this study can be found in Figure 1.

### Single-Crystal and Powder X-ray Diffraction

Attempts were made to grow single crystals in 50:50 w/w ethanol–water mixtures in 5 mL vials at a concentration of 0.2 g/mL. Vials were left at 4 °C in a fridge for several days to create supersaturation and induce nucleation and growth. To test if the diastereomers formed conglomerates or racemates a 1:1 weight equivalent mixture of d- and l-enantiomers was dissolved in the same solvent. Single crystals of sufficient size and quality were measured at −153 °C with an Agilent SuperNova diffractometer equipped with an Atlas CCD detector connected to an Oxford Cryostream low-temperature device, using mirror monochromated Cu Kα radiation (λ = 1.54184 Å) from a Microfocus X-ray source. The crystal structure was solved by intrinsic phasing using the software SHELXT^61^ and then refined by a full matrix least-squares technique based on F2 using SHELXL2014.2.^62^ Powder X-ray diffraction (PXRD) patterns of the bulk crystallized material were obtained using a Bruker D8 venture between 10 and 50° 2θ on a silicon wafer. The software Mercury 3.8^63^ was used to calculate the powder X-ray diffraction patterns for all structures solved and to study and visualize the crystallographic structures and main hydrogen bonding motifs present in the different solid forms. Lattice energy calculations were performed on the solved crystal structures of all compounds using the Visual Habit module in Mercury 3.8.^63^ The Dreiding II Mod force field was selected, and all interactions between a central and its neighboring molecules within a 30 Å radius were estimated and summed up to give the values for the lattice energy in kJ/mol. Lattice energy calculations for all structures were shown to converge within the chosen range of 30 Å.

### DSC Melt Analysis and Phase Diagram Mapping

Single crystals of each enantiomer of xylose and arabinose and those formed from 1:1 mixtures of the enantiomers were ground, and their thermal properties were measured using a Mettler Toledo DSC1^+^. All experiments were performed in a N_2_ environment to gain precise melting points of the analyzed crystals. Heating from ambient temperature to 100 °C was performed at a rate of 10 °C/min. A heating rate of 2 °C/min was used between 100 and 170 °C for the arabinose compounds and between 100 and 160 °C for the xylose. The upper temperatures were chosen to minimize degradation of xylose and arabinose after melting and to better detect the melting point. Nevertheless, sugar decomposition at a high temperature could not be avoided, and recrystallization from melt to study the formed crystal structures was not possible. To work around this, after establishing from X-ray diffraction whether a compound was forming a racemate or a conglomerate, mixtures containing the predicted eutectic structure and an excess of each enantiomer were used for DSC experiments to study the thermal behavior of different enantiomeric compositions. In each sealed aluminum pan, around 10 mg of material was placed inside and flattened to ensure a uniform covering of the pan; three repeats for each measurement were performed. Melting temperatures were calculated from the DSC curves using the Origin Pro 2019 Peak and Onset application with the maximum value of the peak taken as the melting temperature, *T*_m_. The heat of fusion value was obtained by fitting the melting with with a Voigt function, after a baseline subtraction. The “Peak Analyzer” function was used for fitting and integrating such peak to find its area. The area under the curve divided by the mass of the sample represents the heat of fusion value in J g^–1^. Phase diagram mapping was modeled using the Prigogine–Defay and van’t Hoff equations shown below:^50,51^
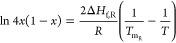
1
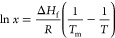
2where *x* is the mole fraction of the enantiomer, Δ*H*_f_ and Δ*H*_f,R_ are the heat of fusion of the enantiomer and the racemic compound respectively, *R* is the universal gas constant, *T*_m_ is the melting temperature of the enantiomer or racemic compound, and *T* is the given temperature in Kelvin.^30,50^

### Solubility Measurements

Solubility measurements were performed via a thermogravimetric method between 5 and 65 °C (with 10 °C increment between each data point) in a 300 mL jacketed vessel. The slurries were heated using a Huber 230 thermoregulator (Huber, Germany) connected to the vessel’s jacket. A vertical condenser was used to reduce the loss of solvent by evaporation during the experiments. Saturated solutions (with extra solid in suspension) were left stirring at each temperature for at least 3 h to ensure equilibration. dl-Arabinose was preslurried for 24 h at 25 °C to ensure that equilibrium conditions were reached. Three repeats of 3 mL injection volume of liquid sample were taken at each temperature; and at high temperatures, the syringes were kept at the same temperature to reduce the possibility of nucleation during sampling. The liquid samples collected in each syringe were passed through a Whatman 0.45 μm filter to ensure that no suspended solid matter was collected into the weighed Petri dish. The solution was left to dry in air at room temperature in weighed Petri dishes, with the initial weight in grams of the Petri dish (*W*_0_), the Petri dish containing the liquid (*W*_e_) and final dry solids (*W*_d_) weights being measured. Solubility was calculated using eq 3.
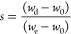
3The solubility of xylose and arabinose solid forms was measured in a solvent mixture of 50:50 ethanol/water at different temperatures. Additionally, solubilities of the same compounds were measured in solvent mixtures of increasing ethanol in 10% w/w increments up to 90% w/w reagent grade ethanol in water at 25 °C.

Equation 4 was used to interpolate the experimental values, where Δ*H*_diss_ and Δ*S*_diss_ are the enthalpy and entropy of dissolution of the solid in the solvent studied.^64^
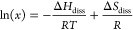
4The experimental solubilities and the thermodynamic parameters extrapolated were compared with the ideal values estimated from thermal data (eq 2).

Activity coefficients were defined as the ratio between ideal and real solubility using eq 5, where γ represents the activity coefficient, expressed as
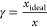
5An average of the activity coefficients over the temperature range 5–55 °C was taken to represent the activity of the compounds in the given solutions of 50:50 and 70:30 ethanol/water. The enthalpy of mixing (Δ*H*_mix_) and the entropy of mixing (Δ*S*_diss_) were determined by their difference from ideal solution behavior through the relationships of eqs 6 and 7.
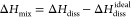
6
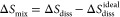
7where the ideal dissolution entropy and enthalpy values (Δ*S*_diss_^ideal^ and Δ*H*_diss_^ideal^) were obtained via interpolating with eq 7 the values of ideal solubilities calculated via eq 2 for each different solid form.

### Slurry Transformation Experiments

Slurry experiments were performed at the 100 mL scale at 25 °C, with stirring via a IKA RCT overhead stirrer (Germany) set at 300 rpm with a R1001 paddle stirrer. A 1:1 mixture of the d- and l-enantiomers of xylose and arabinose was slurried over the course of a week to determine whether they formed racemic compounds and to estimate the kinetics of such transformations. Samples were collected every 2 h, filtered, dried, and measured by PXRD (Cu source, 1.54 Å wavelength) between 10 and 50° (2θ).

## Results and Discussion

### Single Crystals: Microscopy, Morphology, and XRD

**Figure 2:**
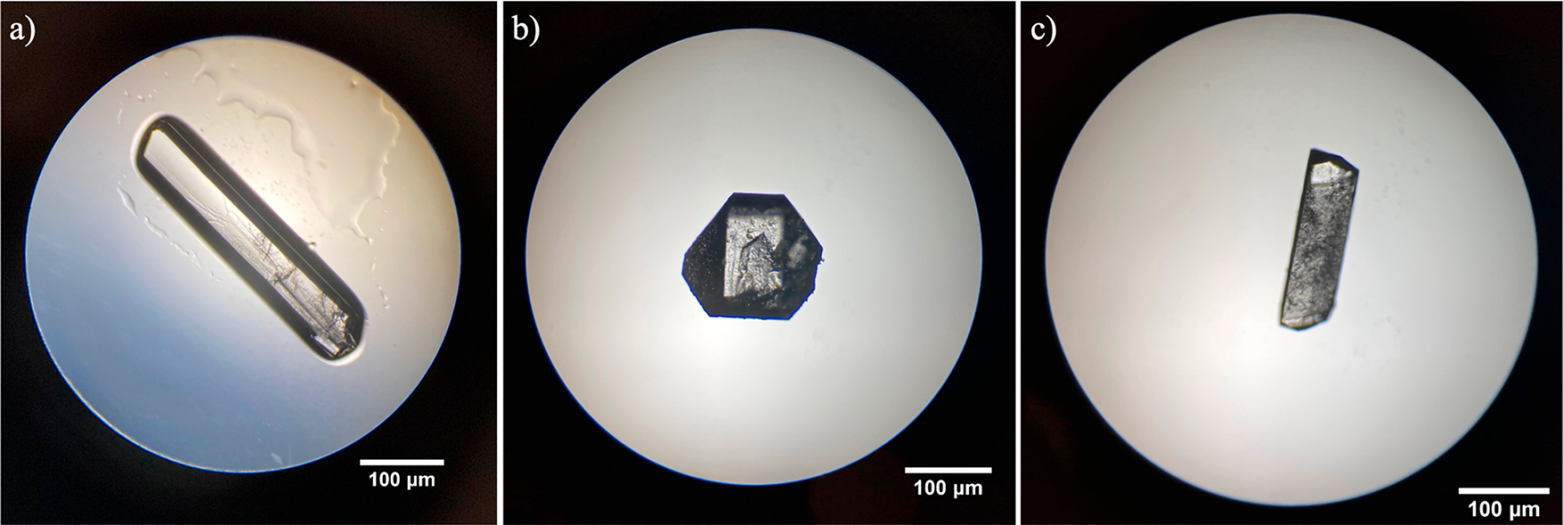
Microscopy images of grown single crystals of d-arabinose (a), dl-arabinose (b), and d-xylose (c). All images taken at 4× magnification.

Single crystals of xylose and arabinose solid forms were grown via slow evaporation from a 50:50 w/w ethanol/water solvent. The crystal structures of l-arabinose, d-xylose, and dl-arabinose were refined with a lower *R* factor compared to the previous literature, whereas structures of the d-arabinose and l-xylose were solved for the first time.^42,46^ Images of the collected single crystals were taken at 4× magnification and showed a difference in morphology between the enantiomeric arabinose forming an elongated rod and the racemic dl-arabinose forming a block shaped crystal. The xylose enantiomers were also shown to form elongated rods (Figure 2a–c).

**Figure 3:**
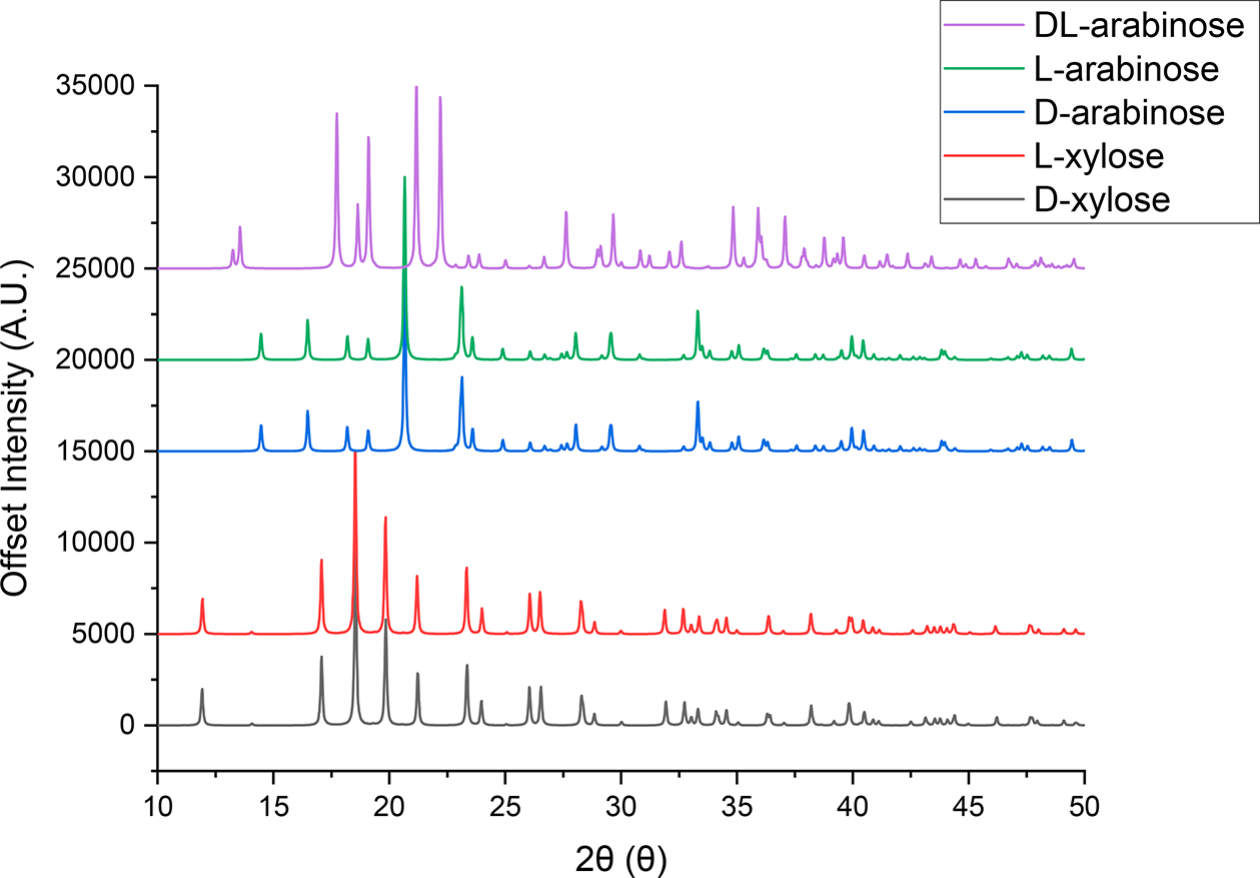
PXRD patterns generated from single-crystal data of d-xylose (black), l-xylose (red), d-arabinose (blue), l-arabinose (green), and dl-arabinose (purple).

Powder X-ray diffraction patterns for all the solved structures were generated from their single-crystal data and are shown in Figure 3. The PXRD pattern of the racemic compound of dl-arabinose displays key characteristic peaks at 14, 15, and 20° (2θ), whereas the key peaks for the d- and l-enantiomer arabinose are at 13, 21, and 22° (2θ). Currently, there are no other known polymorphs or solvated structures for either arabinose or xylose.

**Figure 4:**
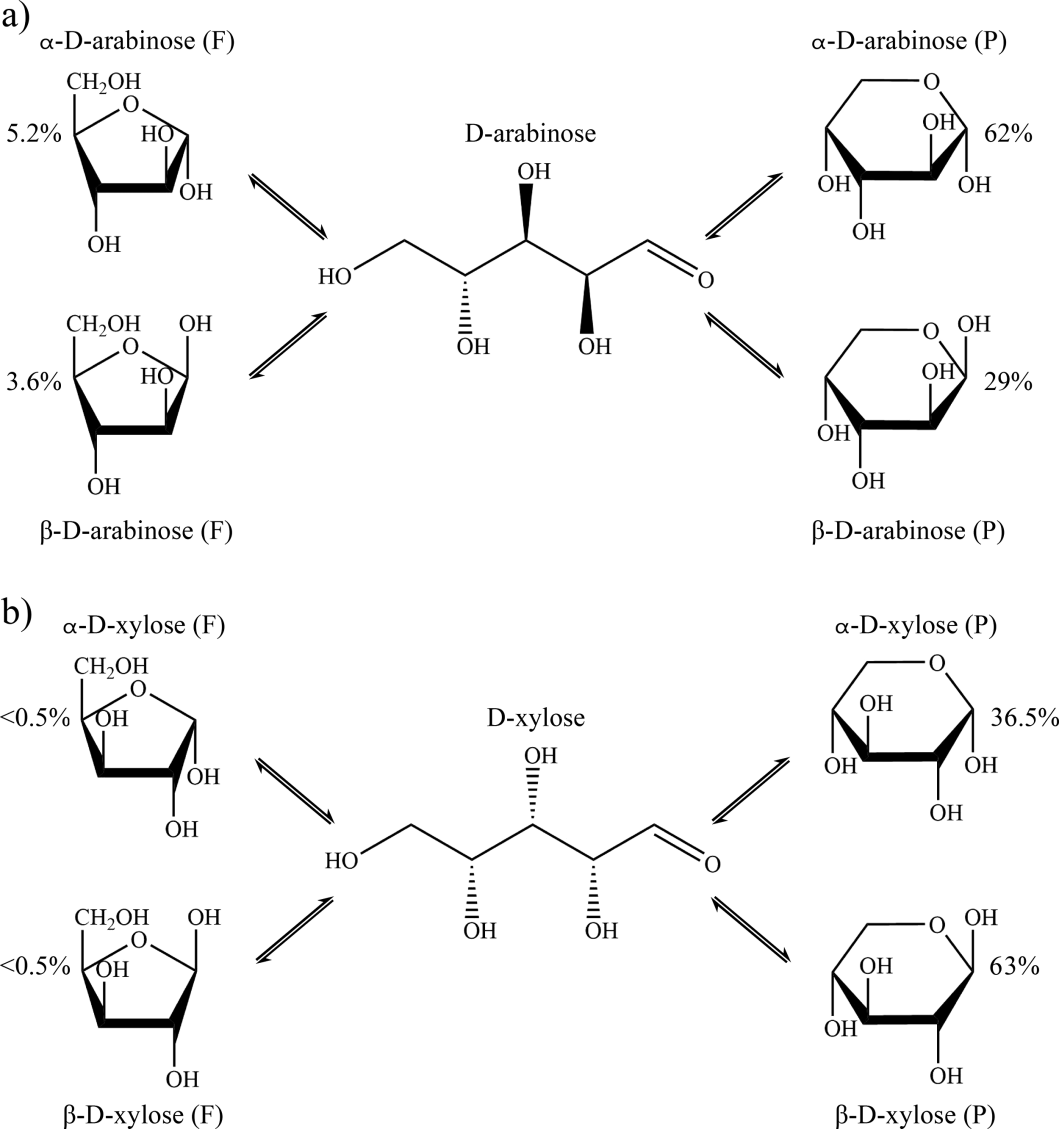
Representations of solution structures with relative amounts in water for (a) d-arabinose at 27 °C^66^ and (b) d-xylose at 31 °C.^44^

As expected with chiral compounds, the unit cell dimensions and angles are the same for each pair of enantiomers, with the only difference being the orientation of the molecules within the lattice.^17,38^ The d- and l-xylose molecules were found to be present in their respective crystal structures as six-membered ring α-anomers,^42,45^ while the d- and l-arabinose crystal structures were made of only six-membered ring β-anomer molecules. The same β-anomer of both d- and l-arabinose molecules was also found in the racemic compound.^2,40^ In order to understand the implications of this crystallization behavior, it is useful to examine the anomeric solution equilibria for xylose and arabinose. As previously mentioned, aldopentose sugars are known to form anomers in solution through ring opening and closing interactions, which establish a dynamic equilibrium. This dynamic equilibrium is dependent on factors such as temperature, pH, and solvent polarity.^44,65,66^Figure 4 shows schematically the structures formed in solution and the anomeric equilibria in water measured for d-xylose at 31 °C (a) and for d-arabinose at 27 °C (b). Both compounds form five-membered rings known as furanose forms (denoted F) and six-membered rings known as pyranose forms (denoted P). While literature data on the solution equilibria are only available for one enantiomer, it is expected that the opposite enantiomer will display a similar ratio of anomers in solution. For both xylose and arabinose, the more abundant six-membered ring molecules crystallize, rather than the five-membered ring forms. However, both xylose and arabinose crystals (including the racemate) contain only one type of the six-membered ring anomers, the less abundant in aqueous solution. This means that the mutarotation equilibrium might have an effect of the kinetics of nucleation and growth for both xylose and arabinose.^45^

It is worth noticing that there are several factors that may affect the equilibria of xylose and arabinose molecules in solution. One of importance to this study is the solvent effect, as mixed aqueous–ethanolic solvent systems were utilized for crystallization. Currently, there are no data available for the anomeric equilibrium of either arabinose or xylose in ethanol; however, their equilibria have been established in DMSO.^66^ It was shown that for arabinose this lower polarity solvent shifted the equilibrium toward the furanose forms at the expense of both the α- and β-pyranose anomer. Conversely, xylose showed a slight increase of the pyranose α-anomer with minimal change to the furanose forms content. This shift in a solvent of lower polarity may lead to changes in the crystallization rates due to the different availability of the crystallizing anomer in solution, as well as the different chemical equilibria and mutarotation rates.^45^

A summary of the key crystallographic information extrapolated from single-crystal XRD analysis can be found in Table 1 for all the crystal structures solved in this work.

**Table 1:** Key Crystallographic Data for d-Arabinose, l-Arabinose, dl-Arabinose, d-Xylose, and l-Xylose

	structure				
property	d-arabinose	l-arabinose	dl-arabinose	d-xylose	l-xylose
CCDC reference number	2114269	2114271	2114270	2114268	2114272
unit cell type	orthorhombic	orthorhombic	monoclinic	orthorhombic	orthorhombic
space group	*P*2_1_2_1_2_1_	*P*2_1_2_1_2_1_	*P*2_1_/*c*	*P*2_1_2_1_2_1_	*P*2_1_2_1_2_1_
unit cell dimensions	*a* = 19.496	*a* = 4.785	*a* = 5.875	*a* = 5.601	*a* = 5.609
	*b* = 6.443	*b* = 6.443	*b* = 7.772	*b* = 9.188	*b* = 9.166
	*c* = 4.783	*c* = 19.490	*c* = 13.249	*c* = 12.574	*c* = 12.590
			β = 99.946		
*R*_1_ (%)	2.30	2.65	3.15	2.35	2.80
anomer conformation	β	β	β	α	α
calculated unit cell density (g/cm^3^)	1.660	1.659	1.674	1.541	1.541
lattice energy (kJ/mol)	–216.5	–211.4	–222.2	–203.5	–202.4

Hydrogen bonding is prevalent within the crystal structure of sugars, due to the high density of hydrogen and oxygen atoms present in this type of molecules.^3,67^ A qualitative analysis of the crystallographic structures solved in this work showed that each different solid form presents different hydrogen bonding motifs. A summary of the main types of hydrogen bonds and their lengths within each structure can be found in Table 2. l-Arabinose was chosen to represent enantiomer arabinose, and d-xylose was chosen to represent enantiomer xylose in all analyses.

**Table 2:** Key Hydrogen Bonding Interactions and Distances for Enantiomer Arabinose, Racemic Arabinose, and Enantiomer Xylose

compound	intermolecular H-bonds between hydroxyl groups bonding distance (Å)	intermolecular H-bonds O to OH bonding distance (Å)
l-arabinose	1.899, 1.972, 2.171	1.951
dl-arabinose	1.880, 1.957, 1.987	1.880
d-xylose	1.808, 1.874, 1.918	1.959

**Figure 5:**
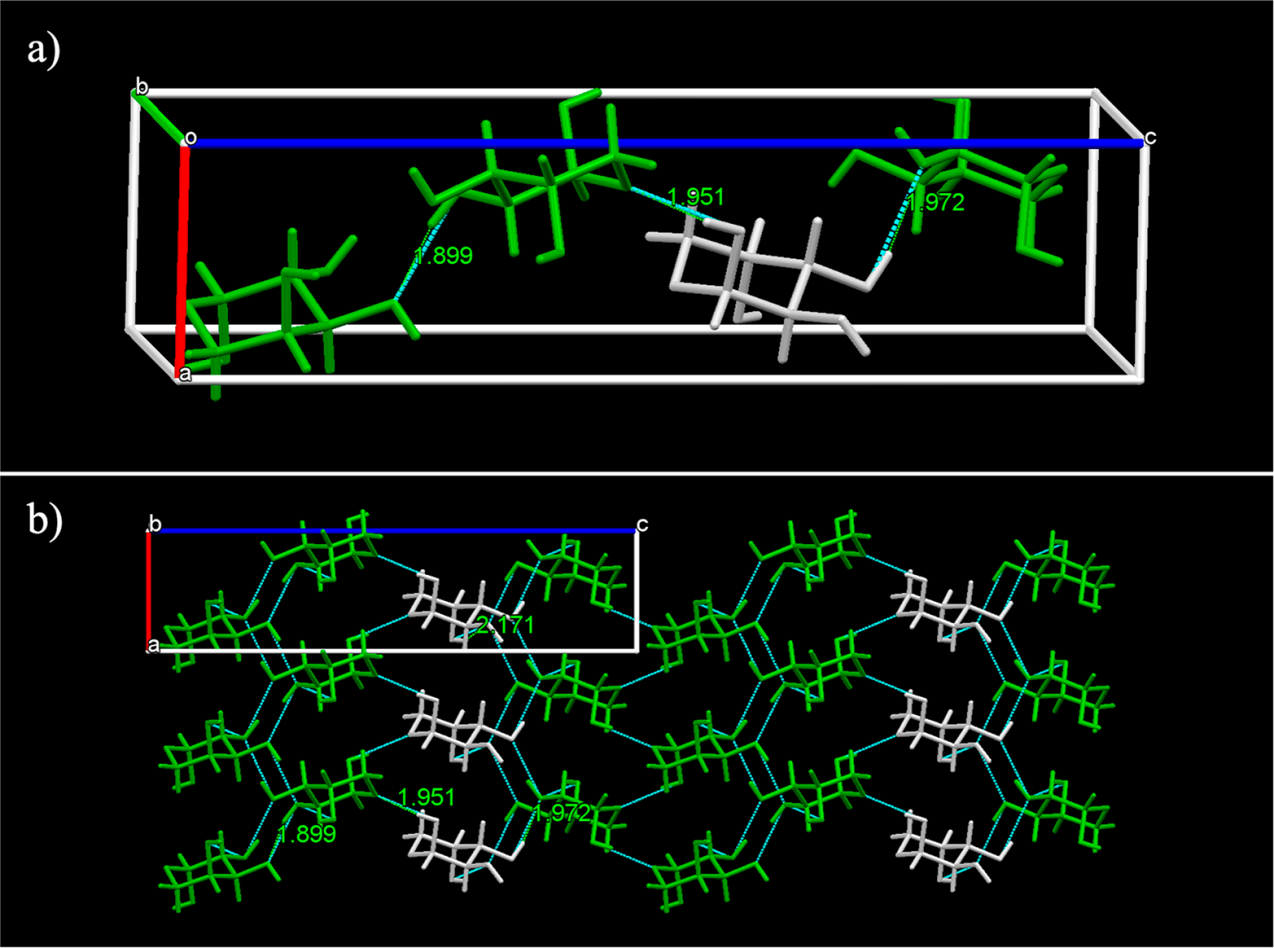
(a) The unit cell of l-arabinose. (b) Packed molecules arranged along the *b* axis with symmetry related screw axis molecules indicated in white and green. H-bonds indicated in dotted blue lines.

The crystal structure formed by β-l-arabinose molecules has an orthorhombic unit cell of space group *P*2_1_2_1_2_1_ that contains four molecules, as shown in Figure 5a. Within the crystal structure, the β-l-arabinose molecules are linked to each other by a network of hydrogen bonds, as shown in Figure 5b. The green molecules are positioned symmetrically to the white (identity) molecules around three different 2_1_ screw axes. The β-l-arabinose molecules give rise to a unit cell of 1.660 g/cm^3^ density, with the longest dimension of the unit cell at 19.49 Å. The lattice energy of l-arabinose is −211.4 kJ/mol. Four types of intermolecular H-bonds can be found in this crystal structure: one between the ring bound oxygen and an OH group at a distance of 1.951 Å, and three bonds between two hydroxyl groups in two different molecules at 1.899, 1.972 and 2.171 Å.

**Figure 6:**
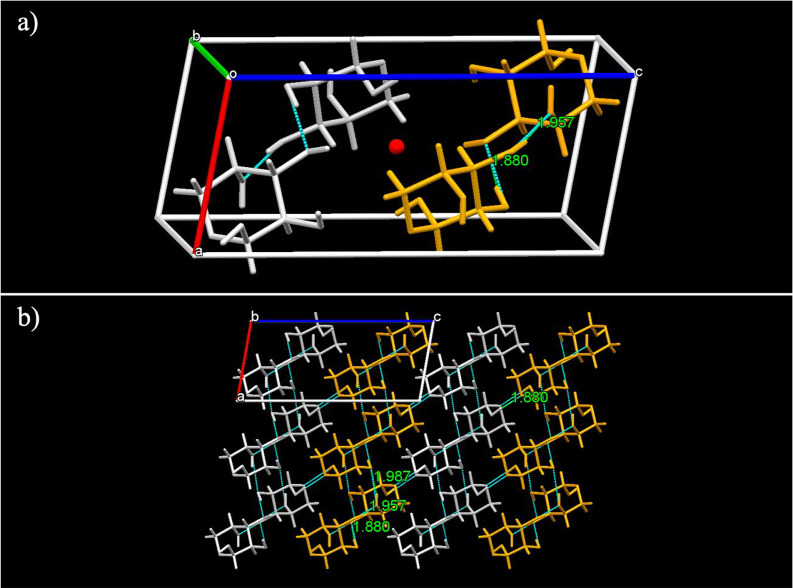
(a) Unit cell of dl-arabinose with d-molecules in gray and l-molecules in orange, showing their hydrogen bonding interactions and inversion center; (b) the packing arrangement of d- and l-molecules along the *b* axis.

The racemic compound of dl-arabinose has as a relatively compact monoclinic structure with a *P*2_1_/*c* space group and only β-anomers of both enantiomers. Within the unit cell, there are four molecules, two pairs of the same enantiomer, each pair placed around an inversion center as shown in Figure 6a, where the β-d-arabinose molecules are in white, while the β-l-arabinose is shown in orange. Both molecules of the same enantiomer occupy the two possible positions of 2_1_ screw axes. The unit cell of the racemic compound has an increased density (1.674 g/cm^3^) compared to the pure enantiomer crystals and a shorter longest edge of 13.25 Å. The lattice energy of the racemic compound is −222.2 kJ/mol, which is lower than the two arabinose enantiomer crystals. This solid form also has a higher density than the pure enantiomers. The relative values of lattice energy and density explain why the racemic compounds form in the presence of both enantiomers. As specified in Table 2, there are four different types of H-bonds within the crystal lattice of the racemic compound: one between the ring oxygen and an OH group, similar to the pure enantiomer crystals but of shorter distance of 1.880 Å, and the others between different hydroxyl groups at distances of 1.880, 1.957, and 1.987 Å, Figure 6b.

As shown in Figure 6b, the d- and l-arabinose molecules are arranged within the racemate structure in layers with a repeating DLLD pattern. Opposite enantiomers within the same layers are linked through intermolecular H-bonds between the ring bound oxygen and hydroxyl groups, while molecules of the same enantiomer, within the same layer and between two different layers, are linked by intermolecular H-bonds between two hydroxyl groups.

**Figure 7:**
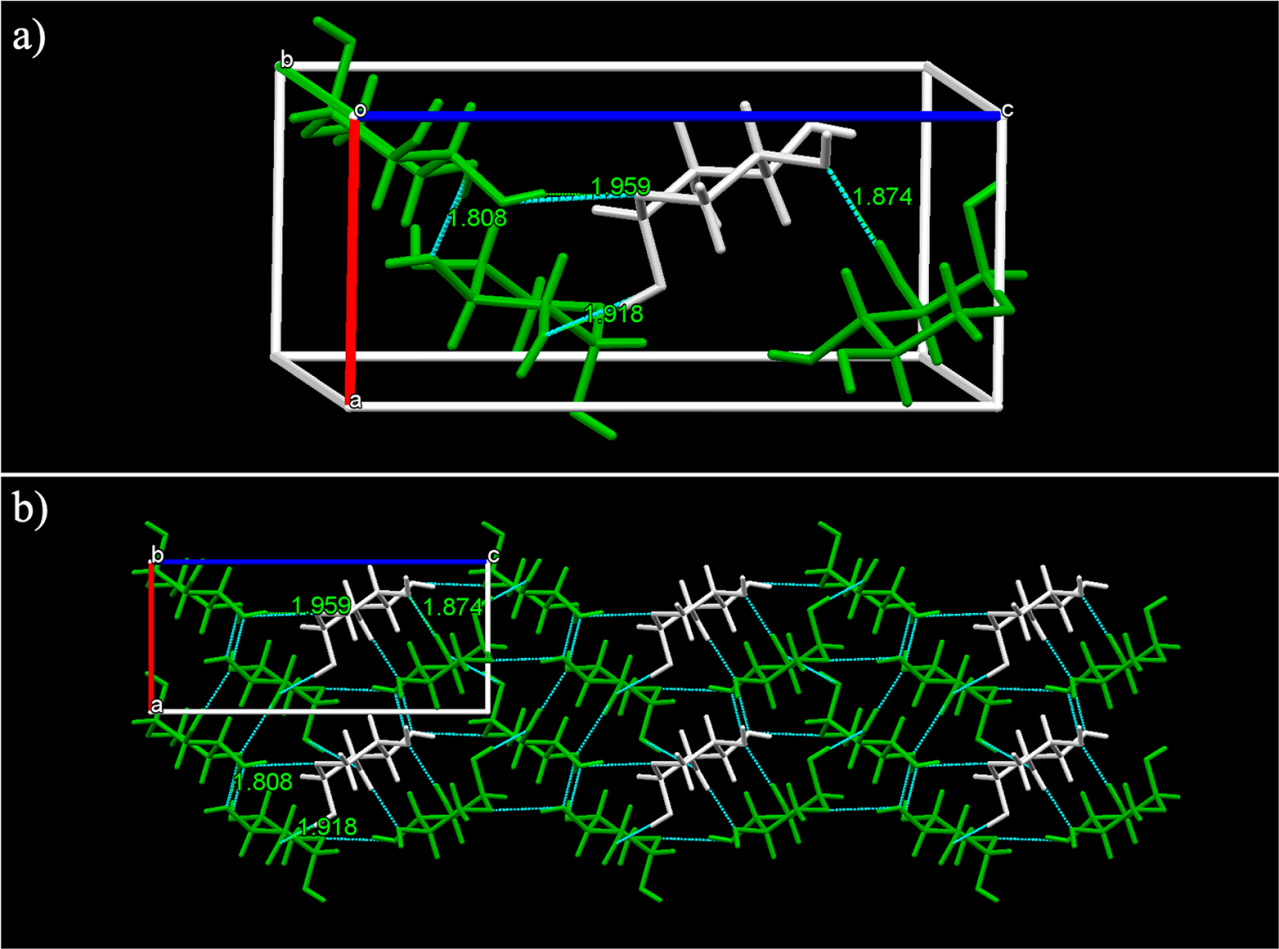
(a) Unit cell of d-xylose showing its screw axis symmetry related molecules (green) and (b) its hydrogen bonding network shown along the *b* axis.

Finally, the d-xylose structure was found to contain only α anomer molecules in an orthorhombic unit cell with a *P*2_1_2_1_2_1_ space group. In Figure 7a, symmetrical molecules with respect to the three different screw axes are indicated with two different colors. This structure has the lowest density (1.541 g/cm^3^) among the ones analyzed, as well as the highest lattice energy of all the solved structures, at −203.5 kJ/mol. The longest edge of the unit cell is 12.57 Å long. α-d-Xylose forms four distinct hydrogen bond types: a ring bound oxygen to a OH group with a distance of 1.959 Å and intermolecular bonds between hydroxyl groups at 1.808, 1.874 and 1.918 Å. The α-d-xylose lattice shows a packing arrangement similar to the β-l-arabinose with the same space group and similar types of H-bonds (Figure 7b).

### Thermal Analysis by Differential Scanning Calorimetry

**Figure 8:**
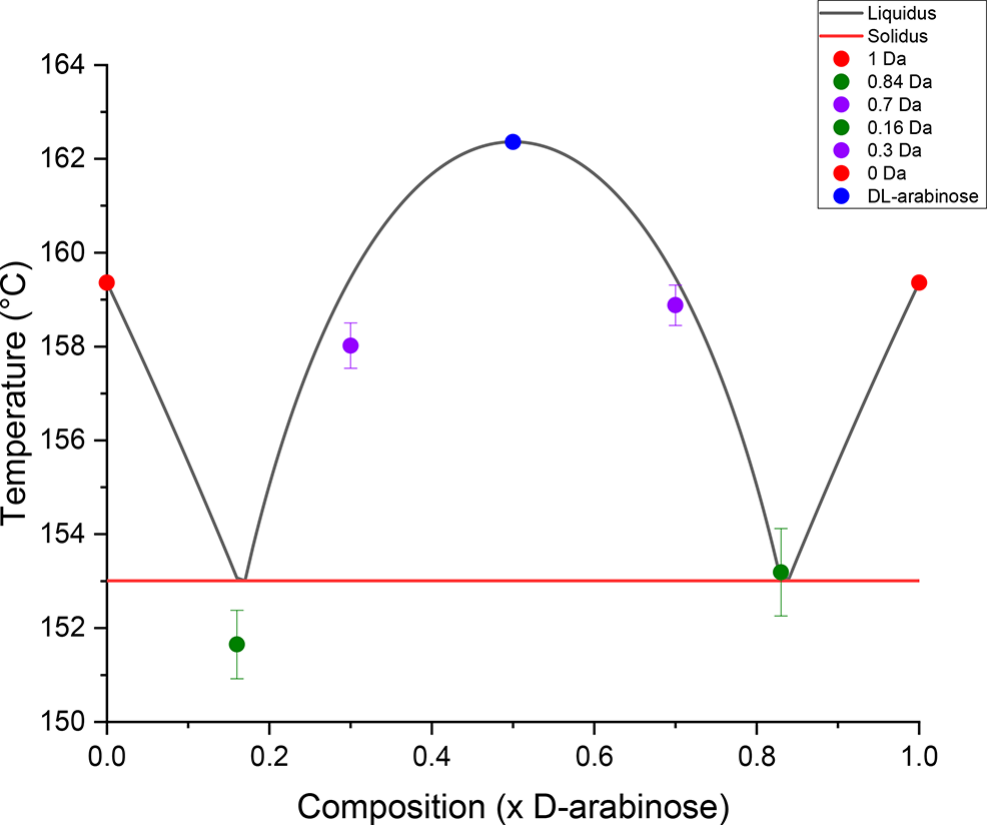
Binary phase diagram of d- and l-arabinose. Liquidus and solidus lines modeled by the Prigogine–Defay and van’t Hoff equations.

Melting studies had previously been conducted with the pure arabinose and xylose enantiomers, but until this work their binary phase diagrams had not been estimated.^68,69^ The main aim of this set of experiments was to map the binary phase diagrams of both arabinose and xylose, rationalizing their crystallization behavior (racemic compound or conglomerate). DSC measurements were conducted using ground samples from single crystals to give the most accurate measure of the melting temperature and the heat of fusion. The melting temperatures of each of the pure compounds and mixtures are shown in Table 3. Good repeatability was achieved for the melting temperatures of the pure arabinose enantiomers and the dl-arabinose racemate. The higher Δ*H*_f_ and *T*_m_ values exhibited by the racemic compound dl-arabinose suggest that it is more thermodynamically stable than the pure enantiomers. This higher Δ*H*_f_ and melting temperature values are in agreement with findings from the lattice energy calculations and the calculated density, both indicating that the racemic compound is more stable. Experimental data also agrees with the modeled results from the Prigogine–Defay equation, assuming that the system forms a racemate. The melting point for the d-arabinose has a relatively higher standard deviation compared to the other analyzed arabinose crystals. This may be due to differences in sample quality; i.e., the crystal may have broad size distribution, which may have led to an uneven distribution of material throughout the pan. The experimental temperatures were within 1.5 °C of the predicted temperature values. The phase diagram for dl-arabinose can be found in Figure 8, with the data points used in Table 3.

**Table 3:** Melting Data from Binary Phase Diagram Construction for dl-Arabinose^a^

material	average *T*_m_ (°C)		average Δ*H*_f_ (J g^–1^)	
d-arabinose	159.97	±0.45	285.6	±20
l-arabinose	158.75	±0.12	279.7	±3.5
average enantiomer	159.36	±0.61	282.7	±14
dl-arabinose	162.36	±0.11	314.1	±2.0

mixture	average eutectic *T*_m_ (°C)		average peak *T*_m_ (°C)	
0.84 (x_*n*_) d-arabinose	153.19	±0.93	N/A	
0.70 (x_*n*_) d-arabinose	155.84	±0.43	158.88	±0.65
0.30 (x_*n*_) d-arabinose	154.37	±0.73	158.02	±0.47
0.16 (x_*n*_) d-arabinose	151.65	±0.48	N/A	

a Melting of the pure enantiomers shown above, and averages of experimentally gained points shown below in the table.

dl-Xylose, which did not form a racemic compound in the single-crystal experiments, was also evaluated by plotting a binary phase diagram. It was found that the pure enantiomers of xylose melt at roughly 12 °C less than arabinose enantiomers. This result is in alignment with the reported lower calculated unit cell density and lattice energy from single-crystal data. Figure 9 shows the experimentally measured points plotted against a van’t Hoff ideal melting behavior for the dl-xylose conglomerate. The experimental trends still agree with the theoretical model, albeit with slightly less accuracy than the arabinose. The 50:50 mixture of d- and l-xylose melts at a eutectic temperature that is over 20 °C lower than the melting temperature of the pure enantiomers, which is expected for conglomerates. The melting temperature of the 50:50 d- and l-mixture is around 1 °C higher than the predicted one, with a low standard deviation of 0.25 °C recorded between the three runs.

**Figure 9:**
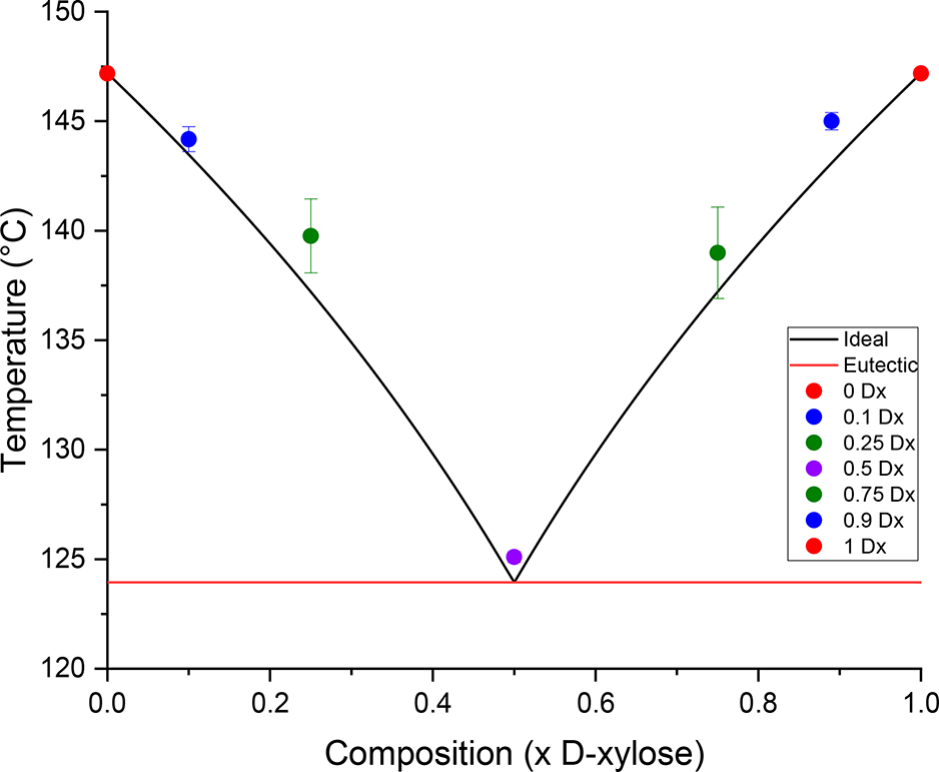
Binary phase diagram of d- and l-xylose in varying proportions. Liquidus modeled by the van’t Hoff equation.

Points measured at 0.1 and 0.9 mole fraction of d-xylose are also close to the expected van’t Hoff model for a eutectic mixture. The experimental points gathered for the 0.25 and 0.75 mole fraction of d-xylose have instead larger standard deviations, perhaps due to uneven distribution of material within the DSC pan.

**Table 4:** Melting Data from Binary Phase Diagram of d- and l-Xylose^a^

material	average *T*_m_ (°C)		average Δ*H*_f_ (J g^–1^)	
d-xylose	148.32	±0.72	273.75	±11
l-xylose	146.04	±0.08	275.52	±1.0
average enantiomer	147.18	±1.14	274.63	±14

mixture	average eutectic *T*_m_ (°C)	
0.90 (x_*n*_) d-xylose	145.00	±0.39
0.75 (x_*n*_) d-xylose	138.99	±2.09
0.50 (x_*n*_) d-xylose	125.10	±0.25
0.25 (x_*n*_) d-xylose	139.76	±1.69
0.10 (x_*n*_) d-xylose	144.18	±0.57

a Melting of the pure enantiomers shown above and averages of experimentally gained points shown below in the table.

The data obtained from the DSC measurements, while confirming the results gained from the study of the single-crystal structures, were essential to obtain the ideal solubilities of every analyzed solid form. These values are shown in the following sections and compared with experimental data.

### Solubility Measurement and Analysis of Thermodynamic Parameters

To further confirm the relative stabilities of the enantiomers and racemic compound for both arabinose and xylose, their solubility was estimated in 50:50 and 70:30 w/w ethanol/water by a thermogravimetric method using eq 3.

**Figure 10:**
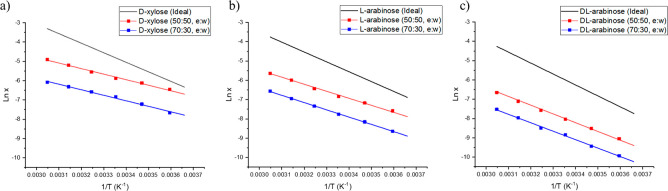
van’t Hoff plots of ideal (black lines) and measured solubility for (a) d-xylose, (b) l-arabinose, and (c) dl-arabinose in 50:50 and 70:30 w/w ethanol/water solvent systems (red and blue lines and points).

The complete data set for d-xylose, l-arabinose, and dl-arabinose are shown in Table 5. The solubility of the racemic dl-arabinose is lower than that of the enantiomer l-arabinose, which indicates that this form is more stable than a mixture of the two enantiomers.^50^ This is in agreement with the structural and thermal analysis shown in the previous sections. Solubilities of all compounds were shown to increase with temperature. d-Xylose showed a much higher solubility than both the arabinose solid forms.^57,59,70^ While it has not been measured experimentally, the solubility of the dl-xylose conglomerate will be double that of a single enantiomer.^71^ This follows the Meyerhoffer’s double solubility rule stating that due to the identical number of d- and l-molecules in solution, the total solubility of the conglomerate will be the sum of the solubilities of the two enantiomers.^72^ Solubilities were also measured at different ethanol concentrations to check the effect of solvent composition, which is essential to determine the optimal conditions that maximize the theoretical yield of crystallization. The complete solubility data for the three solids in 70:30 w/w ethanol/water can be found in Table 5. Experimental solubilities for each compound in the two solvent systems can be found in Figure 10a–c, plotted together with the ideal solubility (represented as a solid black line) determined by the thermal data (eq 2).

**Table 5:** Full Solubility Data for All Species (d-Xylose, l-Arabinose, and the Racemic Compound dl-Arabinose)^a^

solubility in 50:50 ethanol/water (_g_solid/_g_solution)						
temperature (°C)	d-xylose		l-arabinose		dl-arabinose	
5	0.1918	±0.0029	0.0709	±0.0034	0.0173	±0.0003
15	0.2483	±0.0074	0.1027	±0.0122	0.0293	±0.0002
25	0.2954	±0.0052	0.1389	±0.0038	0.0463	±0.0001
35	0.3671	±0.0037	0.1949	±0.0018	0.0713	±0.0009
45	0.4538	±0.0005	0.2735	±0.0014	0.1097	±0.0017
55	0.5257	±0.0117	0.3492	±0.0058	0.1630	±0.0026

solubility in 70:30 ethanol/water (_g_solid/_g_solution)						
temperature (°C)	d-xylose		l-arabinose		dl-arabinose	
5	0.0698	±0.0042	0.0259	±0.0005	0.0072	±0.0007
15	0.1101	±0.0004	0.0414	±0.0019	0.0119	±0.0002
25	0.1616	±0.0024	0.0605	±0.0024	0.0211	±0.0005
35	0.2089	±0.0003	0.0888	±0.0021	0.0299	±0.0015
45	0.2718	±0.0024	0.1253	±0.0020	0.0498	±0.0002
55	0.3417	±0.0018	0.1761	±0.0027	0.0749	±0.0005

a Solubility in 50:50 ethanol/water shown above, and 70:30 ethanol/water below.

While higher ethanol concentration reduced the solubility of all species, this parameter has a greater effect on the arabinose species, reducing their solubility to around 30–35% of the original mixture as opposed to the 65% for xylose. This phenomenon was further investigated by assessing the thermodynamic parameters and activity coefficients for each solution system (Table 6).

**Table 6:** Thermodynamic Parameters and Activity Coefficients (γ) Estimated from the Solubility and Thermal Data for Two Solvent Systems for the Enantiomer Xylose, Enantiomer Arabinose, and Racemic Arabinose

compound	solvent (e/w)	Δ*H*_Diss_ (kJ mol^–1^)	Δ*S*_Diss_ (KJ K^–1^ mol^–1^)	Δ*H*_Mix_ (kJ mol^–1^)	Δ*S*_Mix_ (KJ K^–1^ mol^–1^)	average γ
xylose enantiomer	50/50	23.87	0.032	–17.23	–0.066	3.17 ± 1.29
	70/30	23.79	0.022	–17.31	–0.075	9.58 ± 3.92
arabinose enantiomer	50/50	30.29	0.045	–12.15	–0.055	4.70 ± 1.37
	70/30	31.31	0.041	–11.14	–0.057	12.26 ± 3.29
racemic arabinose	50/50	37.77	0.060	–9.38	–0.048	8.01 ± 1.82
	70/30	36.45	0.048	–10.70	–0.060	19.72 ± 5.09

A thermodynamic analysis was conducted on the measured solubility (Table 6), and the ideal solubility was determined from DSC measurements (Table 7). For all species, the ideal solubilities are higher than the experimental ones, in both solvents and within the temperature range analyzed. This suggests that homogeneous interactions, either between solute–solute or solvent–solvent, are preferred, rather than heterogeneous interactions between solvent and solute in solution.^64^

**Table 7:** Thermodynamic Properties Related to the Ideal Solubilities of Enantiomer Xylose, Enantiomer Arabinose, and Racemic Arabinose^a^

compound	Δ*H*_diss_^ideal^ (kJ mol^–1^)	Δ*S*_diss_^ideal^ (KJ K^–1^ mol^–1^)
xylose enantiomer	41.10	0.098
arabinose enantiomer	42.45	0.098
racemic arabinose	47.15	0.108

a Values obtained from DSC data.

The increase in ethanol concentration resulted in a greater deviation from the ideal behavior for all compounds. The highest change in activity coefficient between the two solvents was observed with d-xylose, which has also the lowest Δ*H*_diss_, indicating its higher solubility compared to the other solid forms. d-Xylose also has the greatest Δ*H*_mix_ term, which implies more favorable mixing in both of the ethanol/water solvent compositions compared to the other diastereomers. It is worth noticing that the ideal values, obtained from DSC measurements, have a much higher standard deviation compared to the thermogravimetric data, which can impact the calculation of the enthalpies and entropy of mixing. The enantiomeric arabinose is the only solid form that presents an increase in Δ*H*_Mix_ with increasing ethanol content. This increase may indicate a greater affinity for water for l-arabinose compared to the other solid forms studied. The dl-arabinose has the highest activity coefficients; however, these seem less affected by changes in the ethanol concentration compared to the other solid forms. The high value of Δ*H*_diss_ for the racemic compound indicates that solute–solute interactions are preferred compared to interactions of the solute with the solvent molecules. This weaker dependency of dl-arabinose solubility on the solvent composition may be of importance for designing crystallization processes.

In fact, as the solubilities for each crystal structure seem to vary differently with the solvent composition, it may be possible to find a solvent mixture that favors crystallization of one specific component from a mixture of the two diastereomers and their enantiomers.

Overall, the thermodynamic data estimated from solubility experiments are in agreement with the previously established thermodynamic analysis, which ranked the dl-arabinose as more stable than the enantiomer arabinose.

### Relative Stability from Slurry Transformation Experiments

**Figure 11:**
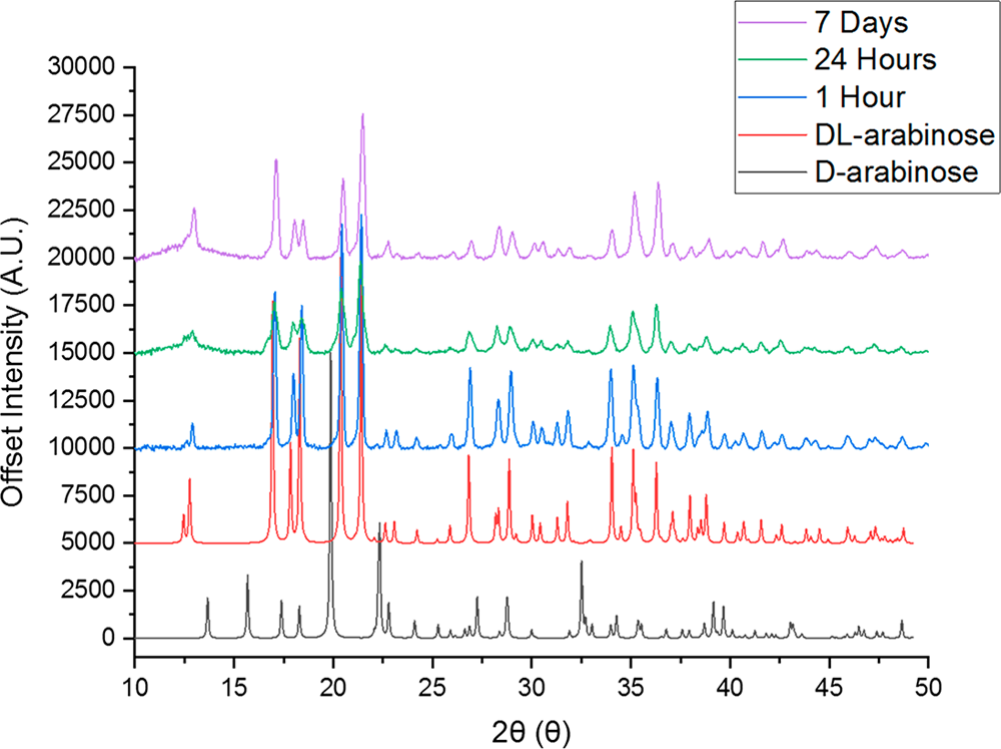
PXRD patterns of the timed slurry transformation from d- and l-arabinose to dl-arabinose (references generated from collected single-crystal data).

As dl-arabinose was shown to form a racemic compound in 50:50 ethanol/water, experiments were carried out to assess the kinetics of the transformation from an enantiomeric mixture into the racemic compound in solution. In order to do this, a slurry of d- and l-arabinose crystals in equal parts was stirred at 25 °C for 1 week. As shown in Figure 11, the conversion from a mixture of enantiomers into the racemic compound occurs within 1 h of experiment. This formation of the racemic compound via slurrying agrees with the considerations made in the previous section and the thermodynamic data. For the mixture of d- and l-xylose, slurrying for 7 days showed no change in the solid form, further indicating that the most stable state for a dl-xylose system is that of a conglomerate.

The slurry experiments confirmed that the most thermodynamically stable form for the dl-arabinose is the racemate, while for the dl-xylose it is the conglomerate. This information is essential for the correct design of processes that can enable the separation of the two enantiomers from a racemic solution. In fact, while the separation of d- and l-xylose enantiomers from a racemic solution could be achieved by crystallization (e.g., seeded preferential crystallization),^36,73^ for arabinose mixtures more complex strategies, such as chiral chromatography, enantiospecific cocrystal formation, or diastereomeric salt formation will be required.^37^

## Conclusions

Microscopy and XRD analysis of single crystals confirmed differences in crystal structure and shape between enantiomers and racemic compounds of xylose and arabinose. The melting behavior of both dl-arabinose and dl-xylose was consistent with the expected behavior for a racemate and a conglomerate, respectively. Solubilities were also measured for two mixed solvents of ethanol/water, which further helped to confirm the relative stability of the different solid forms and their behavior in solution. dl-Arabinose was shown to form a racemic compound that transformed quickly from its constituent enantiomers. This solid form also showed a markedly different crystal structure, particle morphology, melting point, and solubility compared to the pure enantiomers. dl-Xylose, on the other hand, was shown to form a conglomerate, even after prolonged slurrying of a 1:1 mixture of the pure enantiomers. Hence, while preferential crystallization might be an option for the separation of xylose enantiomers from a racemic solution, other strategies will need to be considered for arabinose.

Single crystals for each crystallized form were analyzed via X-ray diffraction and resulted in high-quality data, suitable for a close analysis of the H-bonds motifs and the type of anomer present in each structure. Interestingly, the anomers present in all the xylose and arabinose structures are not the most abundant in aqueous solution, indicating a possible effect of mutarotation equilibrium on the kinetics of crystallization for these two sugars.

Finally, the effect of solvent composition on the solubility of the different xylose and arabinose crystal structures was studied. The results showed that the appropriate choice of a mixed solvent might allow the use of crystallization processes to efficiently separate arabinose and xylose from solutions containing both diastereomers.

Overall, this work provides a crystallographic and thermodynamic analysis of the xylose and arabinose enantiomeric systems, which can be used for the design of more effective unit operations, particularly crystallization, to be used in the extraction of these two natural sugars.

## Accession Codes

CCDC 2114268–2114272 contain the supplementary crystallographic data for this paper. These data can be obtained free of charge via www.ccdc.cam.ac.uk/data_request/cif, or by emailing data_request@ccdc.cam.ac.uk, or by contacting The Cambridge Crystallographic Data Centre, 12 Union Road, Cambridge CB2 1EZ, UK; fax: +44 1223 336033.
